# Using FFPE RNA-Seq with 12 marker genes to evaluate genotoxic and non-genotoxic rat hepatocarcinogens

**DOI:** 10.1186/s41021-020-00152-4

**Published:** 2020-03-30

**Authors:** Chie Furihata, Xinyue You, Takeshi Toyoda, Kumiko Ogawa, Takayoshi Suzuki

**Affiliations:** 1 Division of Molecular Target and Gene Therapy Products, National Institute of Health Sciences, 3-25-26, Tonomachi, Kawasaki-ku, 210-9501 Japan; 2 School of Science and Engineering, Aoyama Gakuin University, Sagamihara, Sagamihara, Kanagawa 252-5258 Japan; 3 School of Public Health, Hongqiao International Institute of Medicine, Shanghai Jiao Tong University School of Medicine, Shanghai, 200025 People’s Republic of China; 4 Division of Pathology, National Institute of Health Sciences, 3-25-26, Tonomachi, Kawasaki-ku, 210-9501 Japan

**Keywords:** FFPE RNA-Seq, PCA, Rat hepatocarcinogen, 2-Acetylaminofluorene, *p*-Cresidine, Genotoxic, Non-genotoxic

## Abstract

**Introduction:**

Various challenges have been overcome with regard to applying ‘omics technologies for chemical risk assessments. Previously we published results detailing targeted mRNA sequencing (RNA-Seq) on a next generation sequencer using intact RNA derived from freshly frozen rat liver tissues. We successfully discriminated genotoxic hepatocarcinogens (GTHCs) from non-genotoxic hepatocarcinogens (NGTHCs) using 11 selected marker genes. Based on this, we next attempted to use formalin-fixed paraffin-embedded (FFPE) pathology specimens for RNA-Seq analyses.

**Findings:**

In this study we performed FFPE RNA-Seq to compare a typical GTHC, 2-acetylaminofluorene (AAF) to genotoxicity equivocal *p*-cresidine (CRE). CRE is used as a synthetic chemical intermediate, and this compound is classified as an IARC 2B carcinogen and is mutagenic in *S. typhimurium*, which is non-genotoxic to rat livers as assessed by single strand DNA damage analysis. RNA-Seq was used to examine liver FFPE samples obtained from groups of five 10-week-old male F344 rats that were fed with chemicals (AAF: 0.025% and CRE: 1% in food) for 4 weeks or from controls that were fed a basal diet. We extracted RNAs from FFPE samples and RNA-Seq was performed on a MiniSeq (Illumina) using the TruSeq custom RNA panel. AAF induced remarkable differences in the expression of eight genes (Aen, *Bax*, *Btg2*, *Ccng1*, *Gdf15*, *Mbd1*, *Phlda3* and *Tubb4b*) from that in the control group, while CRE only induced expression changes in *Gdf15*, as shown using Tukey’s test. Gene expression profiles for nine genes (*Aen*, *Bax*, *Btg2*, *Ccng1*, *Cdkn1a*, *Gdf15*, *Mbd1*, *Phlda3*, and *Plk2*) differed.

between samples treated with AAF and CRE. Finally, principal component analysis (PCA) of 12 genes (*Aen*, *Bax*, *Btg2*, *Ccnf*, *Ccng1*, *Cdkn1a*, *Gdf15*, *Lrp1*, *Mbd1*, *Phlda3*, *Plk2*, and *Tubb4b*) using our previous Open TG-GATE data plus FFPE-AAF and FFPE-CRE successfully differentiated FFPE-AAF, as GTHC, from FFPE-CRE, as NGHTC.

**Conclusion:**

Our results suggest that FFPE RNA-Seq and PCA are useful for evaluating typical rat GTHCs and NGTHCs.

## Introduction

Various ‘omics technologies, including genomics, proteomics, and metabolomics, have been used to evaluate chemical risk assessment. However, in addition to the existing knowledge gaps with regard to linking specific molecular changes to apical outcomes, current methodological uncertainties in interpreting and assessing data limit the application of ‘omics technologies in regulatory toxicology [1].

Targeted mRNA sequencing (RNA-Seq) has become an important tool for examining the role of the transcriptome in biological processes [2]; however, few studies have examined the feasibility of chemical risk assessment using RNA-Seq. Previously, we published RNA-Seq data generated from a next generation sequencer using intact RNA derived from freshly frozen rat liver tissues [3]. We successfully discriminated genotoxic hepatocarcinogens (GTHCs, *N*-nitrosodiethylamine and 3,3′-dimethylbennzidine·HCl) from a non-genotoxic hepatocarcinogen [NGTHC, di(2-ethylhexyl)phthalate] and a different intermediate hepatocarcinogen (1,4-dioxane) [3] using 11 selected marker genes that have been described previously [4]. Recently, Hester et al. described a successful RNA-Seq analysis of formalin-fixed paraffin-embedded (FFPE) tissue [5]. In the present study, we attempted to use FFPE pathology specimens for RNA-Seq. Currently, few papers have been published regarding FFPE RNA-Seq (non-targeted) in the rat liver [6].

The general aims of the present study were to evaluate GTHC and NGTHC via the analysis of selected gene expression patterns within the liver as analyzed using FFPE RNA-Seq and PCA, to determine the usefulness of FFPE RNA-Seq for this analysis, and to compare the typical GTHC, 2-acetylaminofluorene (AAF)-induced gene expression profile to expression profiles that were induced by *p*-cresidine (CRE), which is equivocal for genotoxicity and carcinogenic in rat liver. CRE is used as a synthetic chemical intermediate, and this compound is an IARC 2B carcinogen (rat urinary bladder carcinomas, hepatocellular carcinomas). CRE is mutagenic in *S. typhimurium*, and possesses a weak in vitro ability to affect sister chromatin exchange and to induce chromosome aberrations [7]; however, it is non-genotoxic in rat livers as assessed by single strand DNA damage assays [8].

Previously we proposed the use of 12 mouse marker genes (*Aen*, *Bax*, *Btg2*, *Ccnf*, *Ccng1*, *Cdkn1a*, *Gdf15*, *Lrp1*, *Mbd1*, *Phlda3*, *Plk2* and *Tubb4b*) to discriminate eight mouse GTHCs from four NGTHCs using qPCR [9]. Then, we successfully evaluated these 12 mouse marker genes using publicly available rat toxicogenomic data from the Open Japanese Toxicogenomics Project-Genomics Assisted Toxicity Evaluation System (Open TG-GATEs; https://toxico.nibiohn.go.jp) to discriminate genotoxic from non-genotoxic hepatocarcinogens [4]. The Open TG-GATEs was developed by the Japanese Toxicogenomics Project consortium, analyzed by DNA microarray and opened to the public in 2015 [10]. We compared five rat typical genotoxic hepatocarcinogens (GTHCs) to seven typical non-genotoxic hepatocarcinogens (NGTHCs) and 11 non-genotoxic non-hepatocarcinogens (NGTNHCs; see Methods, Chemicals) at 24 h and 29 days using three doses, and this yielded 124 data points that could be assessed using Open TG-GATEs. Principal component analysis (PCA) of the 12 mouse marker genes successfully separated GTHCs from NGTHCs and NGTNHCs independently of species differences. In the third study, we successfully applied the 12 marker genes to the rat RNA-Seq study with the exception of *Gdf15*, as this gene possessed a low read number in some samples [3]. In the present study, we again applied the 12 marker genes.

As described previously [9], nine (*Aen*, *Bax*, *Btg2*, *Ccng1*, *Cdkn1a*, *Gdf15*, *Mbd1*, *Phlda3* and *Plk2*) of 12 marker genes are members of genes families that are related to the intrinsic apoptotic signaling pathway that is activated by the p53 mediator in response to DNA damage. *Ccnf* may be related to DNA repair and DNA damage [11]. *Lrp1* may modulates cancer progression [12], and *Tubb4b* may be related to human cancer [13].

As described previously [4], we defined the typical rat GTHC as positive according to the standard Ames test and as positive according to in vivo liver tests such as the micronucleus test, the transgenic mutation assay, the comet assay and the UDS test. GTHC were also carcinogenic in rat livers. We defined the typical rat NGTHC as negative according to the standard Ames test and as negative according to in vivo liver tests. These compounds were also carcinogenic in rat livers.

In the present study we successfully extended our RNA-Seq study for use on FFPE samples to discriminate GTHC (AAF) and NGTHC (CRE) via 12 selected marker gene expression patterns in the liver as analyzed using FFPE RNA-Seq and PCA.

## Methods

### Chemicals

#### Chemicals in FFPE RNA-Seq experiment

2-Acetylaminofluorene (AAF, CAS No. 53–96-3) and *p*-cresidine (CRE, CAS No. 120–71-8, IARC 2B carcinogen).

#### Chemicals in PCA calculation from open TG-GATEs data

We calculated five GTHCs, seven NGTHCs and 11 NGTNHCs from Open TG-GATEs (https://toxico.nibiohn.go.jp). The five GTHCs were AAF, aflatoxin B1 (AFL, CAS 1402-68-2, IARC Group 1), 2-nitrofluorene (2NF, CAS 607–57-8, IARC Group 2B), *N*-nitrosodiethylamine (DEN, CAS 55–18-5, IARC Group 2 A) and *N*-nitrosomorpholine (NNM, CAS 59–89-2, IARC Group 2B). The seven NGTHCs were four PPARα agonists [clofibrate (CLO, CAS 637–07-0, IARC Group 3), fenofibrate (FEN, CAS 49562–28-9), gemfibrozil (GEM, CAS 25812–30-0, IARC Group 3) and WY-14643 (WY, CAS 50892–23-4)], two enzyme inducers [hexachlorobenzene (HEX, CAS 118–74-1, IARC Group 2B) and phenobarbital (PHE, CAS 50–06-6, IARC Group 2B)] and ethanol (ETH, CAS 64–17-5, IARC Group 1). The 11 NGTNHCs were allyl alcohol (AA, CAS 107–18-6), aspirin (ASP, CAS 50–78-2), caffeine (CAF, CAS 58–08-2, IARC Group3), chlorpheniramine (CPA, CAS 113–92-8), chlorpropamide (CPP, CAS 94–20-2), dexamethasone (DEX, CAS 50–02-2), diazepam (DIA, CAS 439–14-5, IARC Group 3), indomethacin (IND, CAS 53–86-1), phenylbutazone (PBZ, CAS 50–33-9, IARC Group 3), theophylline (THE, CAS 58–55-9, IARC Group 3) and tolbutamide (TOL, CAS 64–77-7). AAF was registered as a metabolite of 2NF in IARC monograph. FEN, WY, AA, ASP, CPA, CPP, DEX, IND and TOL were not registered in the IARC classification.

### Animal treatment

Fifteen male F344 rats were obtained at 5 weeks of age from Charles River Japan (Yokohama, Japan) and used after 1 week of acclimatization. They were maintained in polycarbonate cages with wood chips as bedding in an air-conditioned room [12-h light (5 a.m. to 5. p.m.), 12-h dark; 23 ± 2 °C; 55 ± 5% humidity] and were provided food (Oriental Yeast Co., Tokyo, Japan) and water ad libitum. The experimental design was approved by the Animal Care and Utilization Committee of the National Institute of Health Sciences, Japan and the animals were cared for in accordance with institutional guidelines and the Guideline for Proper Conduct of Animal Experiments (Science Council of Japan, June 1, 2006). AAF and CRE were purchased from Tokyo Chemical Industry (Tokyo, Japan) and Sigma-Aldrich (St Louis, Missouri, USA), respectively.

Experimental groups (exp) of five male, 6-week-old F344 rats were given chemicals in their food for 4 weeks; AAF: 0.025% and CRE: 1% in food. These doses were similar doses for long-term carcinogenesis studies. Rats in the control group (cont) were given water and a basal diet. After the treatment, the left lobe of the liver was dissected and fixed in 10% formalin for a week, routinely processed to paraffin-embedded blocks, which were used for another study [14], and stored at room temperature for 3 years before use.

### RNA isolation and RNA-Seq

Total RNA was extracted from a liver sample of a FFPE tissue section (10 μm thick with a size range of approximately 200mm^2^) using Maxwell^Ⓡ^ RSC RNA FFPE Kit (Promega Japan, Tokyo, Japan) according to the manufacturer’s instructions. RNA quality was analyzed using an Agilent 2100 Bioanalyzer (Agilent Technologies Japan, Ltd., Tokyo, Japan). RNA-Seq was conducted using TruSeq Targeted RNA Expression Library Prep kits in MiniSeq (Illumina K.K., Tokyo, Japan) according to the manufacturer’s instruction. Approximately 200 ng total RNA from each sample was used for RNA-Seq. The read numbers of each of the 12 genes and *Dazap2* and *Ube2d3* of 15 samples (five each from control, AAF and CRE groups) were analyzed in a single NGS run by adding the oligonucleotide barcode using Local Run Manager Software (Illumina) (Additional file 1). The symbols, gene names and ID numbers of the 12 genes analyzed and *Dazap2* and *Ube2d3* genes, candidate normalized gene are summarized in Table 1. We selected *Dazap2* and *Ube2d3* as candidate normalized genes from public Open TG-GATEs data. (see Discussion in more detail.)

**Table 1:** Fourteen genes analyzed in the present study

No.	Symbol	Gene name	Gene ID
1	Aen	apoptosis enhancing nuclease	361,594
2	Bax	BCL2 associated X, apoptosis regulator	24,887
3	Btg2	BTG anti-proliferation factor 2	29,619
4	Ccnf	cyclin F	117,524
5	Ccng1	cyclin G1	25,405
6	Cdkn1a	cyclin-dependent kinase inhibitor 1A	114,851
7	Gdf15	growth differentiation factor 15	29,455
8	Lrp1	LDL receptor related protein 1	299,858
9	Mbd1	methyl-CpG binding domain protein 1	291,439
10	Phlda3	pleckstrin homology like domain family A member 3	363,989
11	Plk2	polo-like kinase 2	83,722
12	Tubb4b	tubulin beta 4B class IVb	296,554
13	Dazap2	DAZ associated protein 2	300,235
14	Ube2d3	ubiquitin conjugating enzyme E2 D3	81,920

Genes of No. 1–12 were marker genes. Genes of No. 13 and 14 were candidate normalized genes in this study (See Discussion in more detail)

### Statistical analysis

For statistical analysis, we performed a logarithmic (log_2_) transformation of the raw data to stabilize the variance. Statistical significance for each gene against the control group and between AAF and CRE groups was assessed with the Tukey test using “Pharmaco Basic” (Kazuhiko Matsumoto edited, Scientist-press, Tokyo). Differentiation of the gene expression profiles associated with typical GTHCs from those with typical NGTHCs and NGTNHCs and CRE was achieved using the unsupervised learning algorithm PCA [15]. PCA was performed using the PCA program in “R project for Statistical Computing” (https://www.r-project.org/). PCA was conducted with 12 genes (*Aen*, *Bax*, *Btg2*, *Ccnf*, *Ccng1*, *Cdkn1a*, *Gdf15*, *Lrp1*, *Mbd1*, *Phlda3*, *Plk2*, and *Tubb4b*) on FFPE-AAF and FFPE-CRE and our previous Open TG-GATE data (GTHCs, NGTHCs and NGTNHCs) [5].

## Results

### Changes in gene expression in 12 marker genes induced by AAF and CRE

**Fig. 1:**
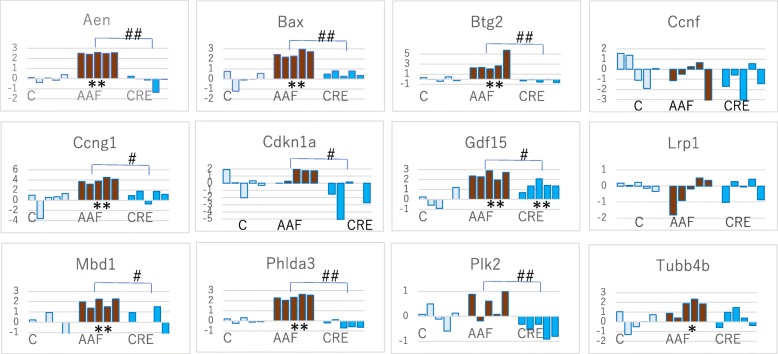
Changes in gene expression in 12 marker genes in the AAF and CRE groups. The mean of each control group was calculated as 0 (log_2_) and ratio (exp/cont) log_2_ was presented. The results of individual rats of control group (C), AAF and CRE were presented. The significance was analyzed using the Tukey test; *, *P* < 0.05; **, *P* < 0.01(each experimental group against the control group). The Tukey test: #, *P* < 0.05, ##, *P* < 0.01 (AAF against CRE)

The read numbers of each of the 12 genes and *Dazap2* and *Ube2d3* genes in 15 samples as assessed using a MiniSeq are provided in Additional file 1. Log_2_-transformed results are presented in Additional file 2. The ratio (exp/cont) log_2_ that was calculated after *Dazap2*-normalization is shown in Additional file 3. Figure 1 presents the bar plots of log_2_-transformed RNA-Seq results (ratio of experimental group/control group in Additional file 3) for the12 genes and the statistical significance of these data were calculated using the Tukey test.

The results demonstrate that significant changes in gene expression in AAF compared to that of the control group were present in eight genes (*Aen*, *Bax*, *Btg2*, *Ccng1*, *Gdf15*, *Mbd1*, *Phlda3* and *Tubb4b*) as determined using the Tukey test; however, CRE induced significant changes only in *Gdf15* expression. The gene expression profiles of the nine genes (*Aen*, *Bax*, *Btg2*, *Ccng1*, *Cdkn1a*, *Gdf15*, *Mbd1*, *Phlda3*, and *Plk2*) were different after treatment with AAF or CRE.

### Discrimination of five GTHCs and FFPE-AAF from seven NGTHCs, 11 NGTNHCs and FFPE-CRE using PCA

**Fig. 2:**
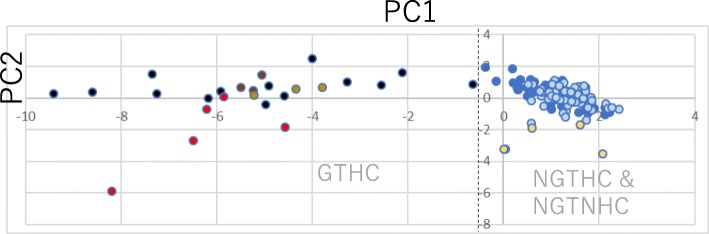
Discrimination of FFPE-AAF from FFPE-CRE together with previous rat GTHCs, NGTHCs and NGTNHCs calculated from public Open TG-GATEs data [4] using PCA. FFPE data show individual results and TG-GATEs data show mean of three rats in each point. Red: FFPE-AAF, brown: AAF at 24 h from Open TG-GATEs, light brown: AAF on 29 days from Open TG-GATEs, black: GTHCs from Open TG-GATEs. Yellow: FFPE-CRE, blue: NGTHCs from Open TG-GATEs, light blue: NGTNHCs from Open TG-GATEs. Two points of FFPE-CRE (− 0.042/− 3.26 and − 0.08/− 3.26) overlapped in Fig. 2. PCA was conducted on data of the ratio (exp/cont) as in Additional file 4 (FFPE-AAF and FFPE-CRE together with previously calculated TG-GATEs data [4]). PC1 and PC2 of the results are shown in Additional file 5. Five typical GTHCs (AAF, AFL, DEN, 2NF and NNM at 24 h and AAF and DEN on 29 days in Open TG-GATEs data) were clearly separated from the seven typical NGTHCs (CLO, ETH, FEN, GEM, HEX, PHE and WY at 24 h and 29 days in TG-GATEs data) and eleven NGTNHCs (AA, ASP, CAF, CPA, CPP, DEX, DIA, IND, PBZ, THE and TOL at 24 h and 29 days in Open TG-GATEs data) using PCA. Two groups of GTHCs and (NGTHCs and NGTNHCs) were separated using PC1 (− 0.637 for DEN24L against − 0.159 for FEN24M. Dashed line is border line of the two groups. FFPE-AAF in GTHCs group was clearly separated from FFPE-CRE grouped in NGTHCs

Log_2_ ratios (exp/cont) of the 12 genes determined from FFPE-AAF and FFPE-CRE samples (Additional file 3) in the present study and previously calculated public DNA microarray Open TG-GATE data for 23 chemicals and 124 data points [five GTHCs (AAF, AFL, 2NF, DEN and NNM), seven NGTHCs (CLO, ETH, FEN, GEM, HEX, PHE and WY) and 11 NGTNHCs (AA, ASP, CAF, CPA, CPP, DEX, DIA, IND, PBZ, THE and TOL)] (Additional file 4) [4] were analyzed using PCA. PC1 and PC2 results are shown in Additional file 5. FFPE-AAF and the five typical GTHCs (AAF, AFL, DEN, 2NF, and NNM) at 24 h and 29 days in the Open TG-GATEs data were clearly separated from FFPE-CRE and the seven typical NGTHCs (CLO, ETH, FEN, GEM, HEX, PHE and WY) at 24 h and 29 days in the Open TG-GATEs data and the 11 NGTNHCs (AA, ASP, CAF, CPA, CPP, DEX, DIA, IND, PBZ, THE and TOL) at 24 h and 29 days in the Open TG-GATEs data according to PCA (Fig. 2). GTHCs were separated from NGTHCs and NGTNHHCs using PC1, where the approximate border line between the two groups was − 0.397 (dashed line in Fig. 2).

## Discussion

High-throughput experiments can be performed using RNA-Seq, as a considerable number of samples and a considerable number of genes can be analyzed simultaneously. RNA-Seq results are highly reliable due to the ability of this method to allow for confirmation of the cDNA sequence. Unlike RNA-Seq, each experiment must be conducted for each sample using standard DNA microarray and qPCR technology, and only relative changes in fluorescent intensity can be measured. Ning et al. also suggested that rapid identification of genetic variants, somatic mutations, gene expression profiles, and epigenetic alterations with single-base resolution can be achieved using RNA-Seq [16].

It was reported that FFPE samples were highly similar to frozen samples with regard to sequencing quality metrics, differentially expressed genes profiles, and dose-response parameters [6]. For the total RNA derived from FFPE samples in the present study, the 260 nm/230 nm ratio was acceptable (approximately 2.0); however, the RNA was short and exhibited a main peak that was below 200 bp. Given that the Illumina short-read sequencer was used and read length for RNA-Seq identification was only 51 bp, the obtained RNA size was sufficient to obtain results. In the present study, we obtained acceptable read number data from FFPE samples compared to that from the previously used freshly frozen samples [3]. We recommend the use of FFPE samples in laboratories, while further methodological development may be required for older lower-quality FFPE samples. Although animal welfare should be considered when performing chemical risk assessment for humans, a 28-day toxicity test in rodents remains essential. Using freshly frozen or FFPE samples from 28 days test for RNA-Seq may prove useful.

The changes in gene expressions in 12 marker genes induced by AAF in FFPE samples that were analyzed by FFPE RNA-Seq in the present study were similar to those in freshly frozen samples that were analyzed by DNA microarray (Open TG-GATEs) [4], and the results for eight of these genes (*Aen*, *Bax*, *Btg2*, Ccng1, *Gdf15*, *Mbd1*, *Phlda3* and *Tubb4b*) were identical. The results indicating.

increases and decreases in the expression of three of these genes (*Ccnf*, *Cdkn1a* and *Lrp1*) were roughly similar. For the PCA data of FFPE-AAF, the average data of five rats in the present study (0.025% in food; approximate 12.5 mg/kg bw/day) were PC1:

− 6.26 and PC2: − 2.25. The PCA data of the Open TG-GATEs of the low dose (30 mg/kg bw/day by gavage, average of three rats) at 29 days were PC1: − 5.23 and PC2: 0.17 (Additional file 5). Numerically, the PC1 score of both experiments were similar, and the PC2 scores both experiments were somewhat different. This Difference may be due to the observed differences in *Cdkn1a* and *Plk2*.

As approximately 90% of human carcinogens are genotoxic carcinogens [15], they remain serious threat to human health. We are developing an in vivo short-term genotoxic carcinogen screening method using gene expression profiles and PCA that is based on the toxic modes of action of various chemical compounds. As a next step, all genotoxic and non-genotoxic carcinogens and non-carcinogens could ideally be screened using gene expression profiles via an in vivo short-term assay.

PCA is an unsupervised learning algorithm. Ringnér wrote that “PCA is often incorporated into genome-wide expression studies.” He explained that “samples can then be plotted, making it possible to visually assess similarities and differences between samples and determine whether samples can be grouped” [17]. In the present study, we demonstrated that PCA analysis of our previous Open TG-GATEs study [4] was useful for predicting the genotoxicity and hepatocarcinogenicity of new chemicals. Here, we have successfully analyzed our FFPE-AAF and FFPE-CRE data against our previous Open TG-GATEs data. The PC1 results for AAF at 24 h and 29 days from Open TG-GATEs data (Additional file 5) were − 5.50 to − 3.79, and the PC1 results for FFPE-AAF (Additional file 5) were − 8.20 to − 4.57. The PC1 results for FFPE-AAF were consistently plotted in the expected areas. Differences in *Cdkn1a* and *Plk2* in the present FFPE data compared to that from our previous Open TG-GATEs data [4] may be responsible for the lower PC2 numerical values obtained in the present study. The PC1 result for DEN24hL (Open TG-GATEs data) was exceptionally close to those of the NGTHCs and NGTNHCs group, and this may be due to the use of a single lower dose of 10 mg/kg bw, as the LD50 of DEN is 220 mg/kg bw by oral administration.

FFPE-CRE was plotted by PCA in the NGTHCs group from Open TG-GATEs data in the present study. Little is known regarding the in vivo genotoxicity of CRE in rat livers. Although CRE is mutagenic in *S. typhimurium* and weakly positive in in vitro sister chromatin exchange and chromosome aberrations [7], the present results and the results of the in vivo rat liver single strand DNA damage test [8] suggest that CRE is not genotoxic in rat livers.

Additionally, we can calculate the approximate value of PC1 as written in the Discussion and Appendix A3 [4]. The first principal component (Y1) is given by the linear combination of the variable X1, X2, −--, Xp.

Y1 = a11X1 + a12X2 + … + a1pXp where a1p is the eigenvector, that can be calculated with the PCA program in R, and Xp is the canonicalized logarithmic (log2)-transformed gene ratio (exp/cont), [(χ-μ)/σ].

χ is the logarithmic log2 of exp./cont, μ is the mean and σ is the standard deviation (https://strata.uga.edu/software/pdf/pcaTutorial.pdf).

When we calculate all 24-h and 29-day data from the Open TG-GATEs data using R, a11, −--, a1p of PC1 are a(Aen): − 0.327, a(Bax): − 0.336, a(Btg2): − 0.324, a(Ccnf): 0.076, a(Ccng1): − 0.344, a(Cdkn1a): − 0.312, a(Gdf15): − 0.312, a(Lrp1): 0.263, a(Mbd1): − 0.207, a(Phlda3: − 0.306, a(Plk2): − 0.313, a(Tubb4b): − 0.243. (See Appendix A.3 in [5]).

Users can calculate their PC1 (Y1) score by introducing their xp into the following equation:

$$ Y1=\left(-0.327\right)\times \left[\left(\upchi \mathrm{Aen}-0.316\right)/0.952\right]+\left(-0.336\right)\times \left[\left(\upchi \mathrm{Bax}-0.320\right)/0.790\right]+\left(-\mathrm{0,324}\right)\times \left[\left(\upchi \mathrm{Btg}2-0.264\right)/0.945\right]+(0.076)\times \left[\upchi \left(\mathrm{Ccnf}+0.0767\right)/0.413\right]+\left(-0.344\right)\times \left[\left(\upchi \mathrm{Ccng}1-0.563\right)/1.16\right]+\left(-0.312\right)\times \left[\left(\upchi \mathrm{Cdkn}1\mathrm{a}-0.405\right)/1.35\right]+\left(-0.312\right)\times \left[\left(\upchi \mathrm{Gdf}15-0.354\right)/1.10\right]+(0.263)\times \left[\left(\upchi \mathrm{Lrp}1+0.094\right)/0.338\right]+\left(-0.207\right)\times \left[\left(\upchi \mathrm{Mbd}1-0.0309\right)/0.344\right]+\left(-0.306\right)\times \left[\left(\upchi \mathrm{Phlda}3-0.211\right)/1.12\right]+\left(-0.313\right)\times \left[\left(\upchi \mathrm{Plk}2-0.172\right)/0.602\right]+\left(-0.243\right)\times \left[\left(\upchi \mathrm{Tubb}4\mathrm{b}-0.276\right)/0.431\right] $$

**Fig. 3:**
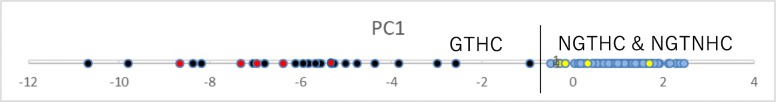
Calculated approximate PC1 of FFPE-AAF and FFPE-CRE with previous rat GTHCs, NGTHCs and NGTNHCs calculated from public Open TG-GATEs data [4] in Additional file 6. Border line between GTHCs and (NGTHCs + NGTNHCs) is − 0.710. Red: FFPE-AAF, black: GTHCs from Open TG-GTAEs [4], yellow: FFPE-CRE (two points of − 0.273 and − 0.304 overlapped in Fig. 3) and blue: NGTHCs and NGTNHCs from Open TG-GTAEs [4]

We show an example of the calculation of AAF-1 in Additional file 6, and we added the approximate PC1 for the FFPE RNA-Seq results in Additional file 6. If users replace the AAF-1 data in Additional file 6 with their own data, they can calculate the approximate PC1 for their data. Figure 3 shows PC1 scores for Open TG-GATEs data and the approximate FFPE RNA-Seq results.

We used *Gapdh* to normalize the gene expression data in our previous studies using DNA microarray, quantitative PCR and RNA-Seq [3, 9, 18–21]. However, the read number of *Gapdh* accounted for 59–79% of the total read number, depending on the increases and decreases of expression in the 11 marker genes used in the previous RNA-Seq study [3]. We assumed that normalization of gene expression profiles to that of the highly expressed gene *Gapdh* would be acceptable, as the linearity of the read numbers was assured according to a wide range of RNA-Seq experiments. In a previous study [3], we analyzed 12 marker genes; however, the results for *Gdf15* could not be used because the read number of *Gapdh* occupied a major portion of the read numbers and the read number of *Gdf15’*s was too low in some samples. In the present study we endeavored to determine a new normalization gene candidate that exhibited lower expression than that of *Gapdh*. We chose *Dazap2* and *Ube2d3* that were selected from the Open TG-GATEs data as the most stable genes (Additional file 7) (DNA microarray data, [https://toxico.nibiohn.go.jp]). These genes did not exhibit significant increases or decreases and their fluorescent expression intensity was approximately 1/3 that of *Gapdh* in the Open TG-GATEs data. We used *Dazap2* as a normalized gene in the present study; however, it was not necessarily an ideal normalized gene in the present study, as the read number for *Dazap2* was 7.53 to12.4% of the total read number in the control group (lower than expected), 7.71 to 14.1% in the CRE group and 2.49 to 5.30% in the AAF group due to the observation that marker genes such as *Aen*, *Bax*, *Btg2*, *Ccng1* and *Phlda3* were remarkably increased in the AAF group. The read number for *Ube2d3* was lower than that of *Dazap2*, and this gene was not suitable as a normalized gene. We will continue to investigate more suitable normalization genes for use in our RNA-Seq analysis.

We previously conducted collaborative studies on toxicogenomics in rodent liver with the Toxicogenomics/Japanese Environmental Mutagen Society • Mammalian Mutagenicity Study Group [9, 18–21]. We began with mouse studies incorporated DNA microarray analyses [18], and we then transitioned to mouse studies [9, 19–21] using qPCR, as this is a highly sensitive technique for detecting and quantifying selected mRNA target [22]. We proposed the use of 12 mouse marker genes [9], and we subsequently applied these 12 mouse marker genes to study rat hepatocarcinogens [3, 4]. The 12 marker genes (*Aen*, *Bax*, *Btg2*, *Ccnf*, *Ccng1*, *Cdkn1a*, *Gdf15*, *Lrp1*, *Mbd1*, *Phlda3*, *Plk2*, and *Tubb4b*) were also useful for discriminating rat GTHCs from NGTHCs and NGTNHCs [3, 4].

## Conclusion

The present results showed that FFPE RNA-Seq of rat liver provides reliable genes expression data comparable with RNA-Seq with fresh frozen tissue. It represents a useful tool for discovery and validation of biomarkers.

## Supplementary information

**Additional file 1:Appendix 1.** Raw read number from 15 samples analyzed by MiniSeq.

**Additional file 2: Appendix 2.** Log2 transferred data.

**Additional file 3: Appendix 3.** Ratio (exp/cont) log2 calculated after Dazap2-normalized.

**Additional file 4: Appendix 4.** Ratio (exp/cont) log2. Open TG-GATEs (DNA microarry) and FFPE-RNA-Seq.

**Additional file 5: Appendix 5.** PC1 and PC2 of FFPE-AAF, FFPE-CRE and previous Open TG-GATEs results [14].

**Additional file 6: Appendix 6.** Gene expression profile (Exp/Cont) at 24 h and 29 days (log2) and calculation of PC1 with the formula.

**Additional file 7: Appendix 7.** selection of control gene-top100 from open TG-GATEs (gene_name, containing Dazap2, Ube2d3 and Gapdh).

## Data Availability

All data generated or analyzed during this study are included in this published article.

## Abbreviations

AAF: 2-acetylaminofluorene
CRE: *p*-cresidine
GTHC: Genotoxic hepatocarcinogen
IARC: International Agency for Research on Cancer
NGTHC: Non-genotoxic hepatocarcinogen
NGTNHC: Non-genotoxic non-hepatocarcinogen
PCA: Principal component analysis
QPCR: quantitative real-time PCR
RNA-Seq: Next generation sequencing-targeted mRNA sequence
